# SisFall: A Fall and Movement Dataset

**DOI:** 10.3390/s17010198

**Published:** 2017-01-20

**Authors:** Angela Sucerquia, José David López, Jesús Francisco Vargas-Bonilla

**Affiliations:** SISTEMIC, Facultad de Ingeniería, Universidad de Antiquia UDEA, Calle 70 No. 52-21, 1226 Medellín, Colombia; angels1031@gmail.com (A.S.); jesus.vargas@udea.edu.co (J.F.V.-B.)

**Keywords:** triaxial accelerometer, wearable devices, fall detection, mobile health-care, SisFall

## Abstract

Research on fall and movement detection with wearable devices has witnessed promising growth. However, there are few publicly available datasets, all recorded with smartphones, which are insufficient for testing new proposals due to their absence of objective population, lack of performed activities, and limited information. Here, we present a dataset of falls and activities of daily living (ADLs) acquired with a self-developed device composed of two types of accelerometer and one gyroscope. It consists of 19 ADLs and 15 fall types performed by 23 young adults, 15 ADL types performed by 14 healthy and independent participants over 62 years old, and data from one participant of 60 years old that performed all ADLs and falls. These activities were selected based on a survey and a literature analysis. We test the dataset with widely used feature extraction and a simple to implement threshold based classification, achieving up to 96% of accuracy in fall detection. An individual activity analysis demonstrates that most errors coincide in a few number of activities where new approaches could be focused. Finally, validation tests with elderly people significantly reduced the fall detection performance of the tested features. This validates findings of other authors and encourages developing new strategies with this new dataset as the benchmark.

## 1. Introduction

The number of elderly people living alone has been continuously growing worldwide. This independence comes with the risk of not receiving prompt attention if an accident occurs. A third of the people over 65 years old suffer on average one fall per year [1], and this number grows with age [2] and previous falls, where about one third develop fear of falling again [3,4]. Not receiving attention in the first hour of the accident increases the risk of death and chronic affections [5]. This issue has been widely addressed in recent years with systems that detect falls in elderly people, and generate a prompt alert that can reduce the consequences related to medical attention response time [6]. These systems have acceptance among the objective population as a way to support their independence and reduce their fear of falling [7].

Developers of fall detection systems are currently facing several challenges. Independently of the acquisition strategy, most works are not tested with the objective population (elderly people), reducing their accuracy in real-life applications [8]. Moreover, all public datasets exclusively contain data from young adults, making it difficult to test new proposals [9]. Here, we make publicly available a dataset with falls and activities of daily living (ADLs) acquired with a wearable device, and we provide results of some of the most commonly used detection features with both young and elderly people. The purpose of this work is to provide a benchmark for other researchers on the fall and movement detection field, and to address two rarely discussed open issues: training with young people features intended for elderly people, and setting-up algorithms for maximum accuracy instead of maximum sensitivity.

Falls are commonly detected with wearable or ambient-based systems (see [6,9,10,11,12] for reviews in the field). Ambient-based sensors such as cameras are intrusive and do not solve the problem for independent adults, who are not confined to closed spaces. According to [2], up to 50% of the falls in independent elderly people occur outside the home premises. Wearable devices offer portability as they can be used regardless of the user location. Available wearable devices include smartphone apps and self-developed systems. In both cases, the preferred sensor is the triaxial accelerometer because of its low cost, small size, and because it is built-in in almost all smartphones [6]. Smartphones are a popular selection for authors because they include a robust hardware, a powerful processor, and they are economically affordable [6,11]. However, the low cost of the individual components and design tools has encouraged authors to develop their own embedded devices too [13].

Independently of the device used, authors have faced problems such as energy consumption, battery life, false positives (the alarm turns on with ADL), false negatives (the alarm does not turn on with falls), and user comfort issues. Specifically for smartphones, these devices are not designed for constant use of the processor and sensors (the battery goes off in a couple of hours [11]). Additionally, the smartphone may get hits and falls caused by manipulation, or the person may forget it in a table after calling, making it less feasible for permanent monitoring.

New strategies for solving the aforementioned problems require testing. It requires acquiring datasets with common types of falls and ADLs. In this sense, some authors analyzed how elderly people fall. Back in 1993, authors in [5] performed a wide survey with 704 women over 65 years old. They reported that most falls were caused by trips, slips and loss of balance. However, they did not record data. About the conditions of the fall, in [14], the authors found that women were three times more likely to hit the ground on the hips than men, and that most people fell in a forward direction with 60% of prevalence. Most activities currently selected for testing algorithms are based on these studies.

Once the selected ADL and falls are simulated and recorded, the raw acceleration data must be processed and classified. Authors commonly filter the data, apply a feature extraction, and classify activities as falls or ADL. The literature provides a wide number of features (see [9], Table 4 and [12], Table 2 for complete lists). Unfortunately, there are not works in the literature tested with independent elderly people (see [9], Table 1, and [12], Table 4) and available public datasets were all recorded exclusively with young adults. In [8], for example, authors tested 13 state-of-the-art approaches with real elderly people falls, and they found that the performance of these approaches severely decreased under real-life conditions. However, they did not release the validation dataset, i.e., other authors cannot analyze why those features reduced their performance, and, more importantly, how to solve it. To our knowledge, there only exist four public datasets, all acquired using smartphones: Mobifall [15], tFall [16], DLR [17], and project gravity [18]. Igual et al. [19] compared the former three and found severe variability and performance issues of the analyzed algorithms.

In this paper, we present and make publicly available a complete dataset of falls and ADL acquired with a self-developed embedded device that can be easily replicated (see [20,21] for example designs). It includes young adults and elderly people performing a wide variety of activities selected from a survey and previous studies. The dataset contains 19 types of ADLs and 15 types of falls. It includes acceleration (from two accelerometers) and rotation (from a gyroscope) data from 38 volunteers divided into two groups: 23 adults between 19 and 30 years old, and 15 elderly people between 60 and 75 years old. The dataset is available for free download as Supplementary Materials [22], and videos of each type of activity within the dataset are also included for helping the reader to replicate this work. Additionally, a comparative analysis between several features used in the literature is presented as a reference for future works.

## 2. Related Public Datasets

Our search on public datasets of falls and ADLs was focused on wearable devices, as ambient based and video fall detection systems are too restrictive to help independent elderly people (the objective population of this work). We also considered some basic requirements for a dataset to be useful: all activities must be well documented, the raw data must be freely available, the dataset must contain both falls and ADLs ([23], for example, examining several public datasets, but none including falls), and it must be reported in a peer-reviewed paper. Following these premises, we found four datasets:

- MobiFall [15]: twenty-four volunteers (22 to 42 years old) performed nine types of ADLs and four of falls using a Samsung Galaxy smartphone, Samsung, Seoul, South Korea. Nine subjects performed falls and ADLs, while 15 performed only falls (three trials each).
- tFall [16]: ten participants between 20 and 42 years old. They recorded eight types of falls (503 total recordings with two smartphones), and one week of continuous ADL recordings with all participants carrying smartphones in the pockets and a handbag. The ADL trials were not identified by activity.
- DLR [17]: sixteen subjects (23 to 50 years old). They recorded six types of ADLs, and the authors did not specify the conditions of the falls (they belong to a single group). The files are too short for some types of analysis.
- Project gravity [18]: three participants (ages 22, 26, and 32) performed 12 types of falls and seven types of ADLs with a smartphone in the pocket.

None of these datasets includes elderly people, and their variety of activities and number of subjects is limited compared to this work. Additionally, all authors used smartphones in the pocket for recordings. Here, we fixed our device as a belt buckle as recommended in previous works [24,25].

## 3. Materials and Methods

### 3.1. Selection of Activities

Additionally to those falls and ADLs commonly tested in the literature (see [9], Table 4), we performed a survey with elderly people living alone and administrative personnel from retirement homes. The survey consisted of three main questions: For each fall incident, (*i*) which activity were you performing when the fall happened? (*ii*) What produced the fall? A sliding, a faint, a trip, other? (*iii*) In which orientation did the fall happen? What part of the body received the impact?. The survey was conducted with 15 elderly people from the psycho-physic program of the Universidad de Antioquia (between July and August 2014, in Medellín, Colombia), and 17 retirement homes (between October 2014 and January 2015, in Medellín and Manizales, Colombia).

As a result of the survey, the independent elderly people fall more when walking, taking a shower, and walking up or down stairs; and fall less when trying to get up or sit down in a chair or a bed, or bending. On the other hand, elderly people living in retirement homes fall more when walking and when trying to get up from a chair or a bed and fall less when walking up or down stairs. The answers given by the participants were consistent with the results presented in [5]. Table 1 shows the types of falls selected for this work. Falling when walking up or down stairs was identified as a common type of fall in the survey, but it was not included here because of the high risk of having an accident.

ADLs of Table 2 were selected based on: common activities, activities that are similar (in acceleration waveform) to falls, and activities with high acceleration that can generate false positives. All ADL and falls selected for this work were approved by a physician specialized in sports. The Supplementary Materials contains videos of each type of fall and ADL performed by the participants, as an effort to solve another drawback in the literature: showing the exact conditions of the recordings [22].

### 3.2. Participants

This database was generated with collaboration of 38 volunteers divided into two groups: elderly people and young adults. Elderly people group was formed by 15 participants (8 male and 7 female), and the young adults group was formed by 23 participants (11 male and 12 female). Table 3 shows age, weight, and height of each group. Individual information is available in the readme [22]. The elderly people group is formed by retired employees of the Universidad de Antioquia and parents of current employees. They all were healthy and independent, and none of them presented gait problems.

Young adults performed ADLs and falls. Elderly people did not perform falls and activities D06, D13, D18, and D19 from Table 2 due to recommendations of the physician specialized in sports. Additionally, some elderly people did not perform some activities due to personal impairments (or medical recommendation). The participant of 60 years old identified by code SE06, who is an expert in Judo simulated both falls and ADLs.

All subjects gave their informed consent for inclusion before they participated in the study. The study was conducted in accordance with the Declaration of Helsinki, and the protocol was approved by the Bio-Ethics Committee of the Medicine Faculty, Universidad de Antioquia UDEA (Medellín, Colombia). Additionally, all participants were evaluated by a physician specialized in sports.

### 3.3. Experimental Set-Up

**Figure 1:**
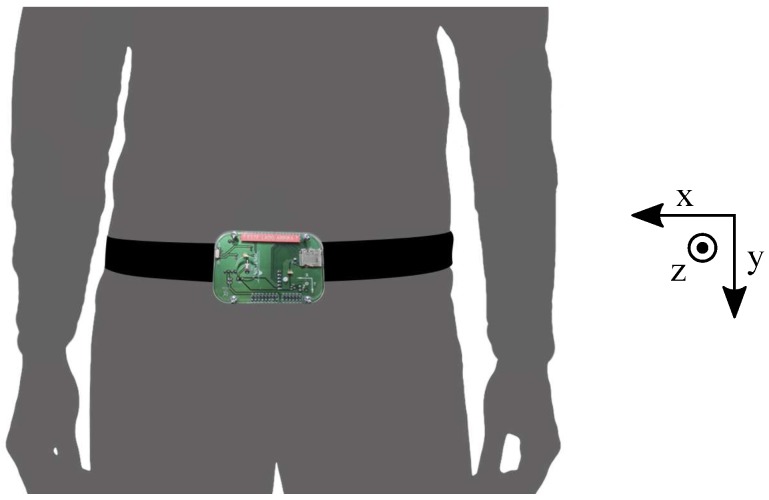
Device used for acquisition. The self-developed embedded device included two accelerometers and a gyroscope. It was fixed to the waist of the participants.

The dataset was recorded with a self-developed embedded device composed of a Kinets MKL25Z128VLK4 microcontroller (NPX, Austin, Texas, USA), an Analog Devices (Norwood, Massachusetts, USA) ADXL345 accelerometer (configured for ±16 g, 13 bits of analog to digital converter –ADC), a Freescale MMA8451Q accelerometer (±8 g, 14 bits of ADC), an ITG3200 gyroscope ($\pm 2000^{\circ }$/s, 16 bits of ADC. Texas Instruments, Dallas, Texas, USA), an SD card for recording, and a 1000 mA/h generic battery. The device was fixed to the waist of the participants (Figure 1). This location provides high distinction among activities for a single accelerometer system [24,25].

Only acceleration data acquired with the ADXL345 sensor was used in this work, as it is energy efficient and provides the larger span. However, the data recorded with the other accelerometer and the gyroscope are also publicly available for further studies. The orientation of the sensor (see Figure 1) presents the positive *z*-axis in the forward direction, the positive *y*-axis in the gravity direction, and the positive *x*-axis pointing to the right side of the participant. All tests were performed with the original frequency sample of 200 Hz.

The classrooms and open spaces of a coliseum at the Universidad de Antioquia (Medellín, Colombia) were used for recording the activities. In order to guarantee safety conditions, falls were simulated using safety landing mats. Activity D17 from Table 2 was recorded using the copilot chair of a Renault Logan car. The time required for recording all trials was approx. 1.5 h for each elderly person and 3.5 h for each young adult.

### 3.4. Fall Detection Algorithms

Here, we test commonly known features as a way to provide a preliminary analysis with the proposed dataset. We follow the common pipeline to process the data: preprocessing, feature extraction, classification, and validation.

#### 3.4.1. Preprocessing Stage

Preprocessing is critical in the performance of the classification algorithms and their computational burden. In this work, we performed a comparison between using preprocessing or not in fall detection. The preprocessing stage consisted of a 4th order IIR Butterworth low-pass filter with cut-off frequency of 5 Hz. This filter was selected due to its simplicity, as it presented similar results than more elaborated IIR and FIR filters (including different cut-off frequencies) that we analyzed in preliminary tests.

#### 3.4.2. Feature Extraction

The objective of this stage is to maximize the separation between ADL and falls. We tested several commonly used features listed in ([9], Table 4 and [12], Table 2) (original implementation details can be followed in the references therein). We separated the features in five groups: amplitude, orientation angle, statistical moments, critical phase time, and area under the curve. Table 4 includes those features that presented the best overall performance.

Here, one sample of acceleration in the three axis is defined as the vector $\vec{a}={\left[a_{x},a_{y},a_{z}\right]}^{T}\in {ℜ}^{3}$, the sliding window used for computing the dynamic features is denoted with $\tilde{a}\left[k\right]={\left[{\vec{a}}^{T}\left[k-N_{v}+1\right],\cdots ,{\vec{a}}^{T}\left[k\right]\right]}^{T}\in {ℜ}^{N_{v}\times 3}$, at time sample *k*, where $N_{v}$ is the number of samples in the selected window. The standard deviation operator is defined as σ(·), and RMS refers to the Root Mean Square value. The integrals were computed with the trapezoid method, with limits $k-N_{v}+1$ to *k*.

#### 3.4.3. Classification

A simple to implement threshold-based classifier was selected for this work. Threshold-based classification is still the most widely used strategy for fall detection, as it is less computationally intensive than support vector machines and similar classification algorithms [11]. We analyzed two widely used alternatives: Threshold 1 ($T_{1}$) which follows maximum accuracy, and Threshold 2 ($T_{2}$) which maximizes the sensitivity (fall detection capability). The sensitivity (SE), specificity (SP) and accuracy (AC) were computed as follows [26]:

(1) $$\mathrm{SE}=\frac{TP}{TP+FN}\;\mathrm{SP}=\frac{TN}{TN+FP}\;\mathrm{AC}=\frac{\mathrm{SE}+\mathrm{SP}}{2},$$

where TP and TN are the true positives and negatives; FP and FN the false positives and negatives, respectively. The way we computed the accuracy allows using an unbalanced number of ADLs and fall trials in a single test. Validation data was tested with the chosen thresholds following a 10-fold cross-validation.

**Figure 2:**
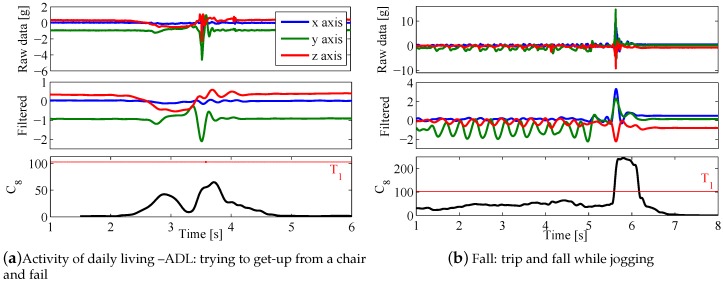
Example of processing and classification. The features are computed after the filtering process of the raw data. (**a**) ADL D11 gives $C_{8}$ values below threshold $T_{1}$ (horizontal **red** line); (**b**) Feature $C_{8}$ crosses the threshold when the fall in activity F05 is detected.

Figure 2 shows an example of the preprocessing stage and the computation of feature $C_{8}$ for ADL D11 (trying to get-up from a chair and fail—Figure 2a) and fall F05 (trip and fall while jogging—Figure 2b), with threshold $T_{1}$. This ADL was selected because of its high peak acceleration. Despite this, $C_{8}$ peak was around 40% below the threshold value (Figure 2a—bottom). On the other hand, feature $C_{8}$ far crossed the threshold during fall F05 (Figure 2b—bottom). Note that while jogging before the fall, which is a high acceleration activity, feature $C_{8}$ was always below the threshold.

#### 3.4.4. Cross-Validation

The robustness of the classification stage was analyzed with a 10-fold cross-validation set-up. All analysis were performed guaranteeing the same proportion of falls and ADLs in the groups. Each group was used in one fold as validation data.

In the following section, we analyze three commonly discussed issues: the effect of preprocessing, the importance of including elderly people in the training stage, and the way the threshold is selected. We finish this study with a novel activity-by-activity analysis that demonstrates how most errors occur in specific activities.

## 4. Results

### 4.1. Effect of Filtering as the Preprocessing Stage

**Figure 3:**
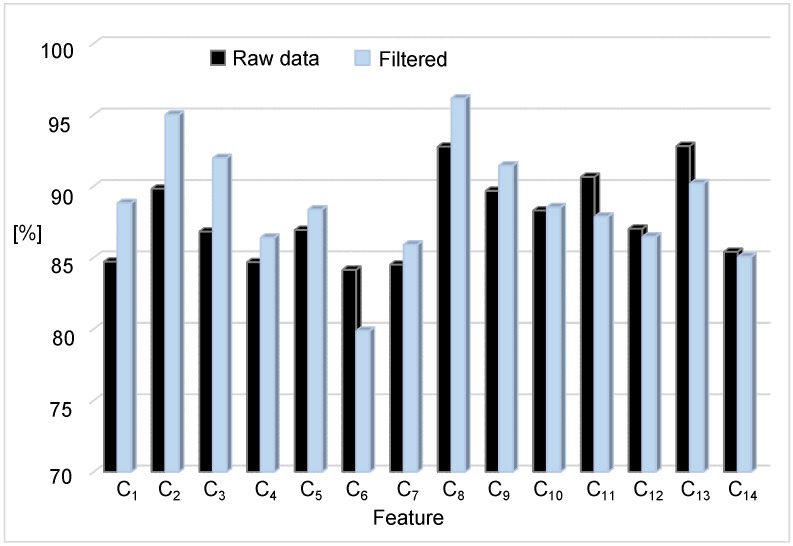
Accuracy obtained in validation after a 10-fold cross-validation without (raw data) and with preprocessing (filtered). Features $C_{2}$ and $C_{8}$ achieved 95.0% and 96.1% of accuracy when the filter was applied, respectively. However, not all features improved their performance after filtering.

We initially tested the effect of filtering before applying the features of Table 4. We used data from all 38 subjects for this analysis (4510 trials). Figure 3 shows the mean accuracy obtained in validation with each feature after a 10-fold cross-validation for both raw and filtered data. Dynamic features were computed within sliding-horizon windows with full overlap. The window size ($N_{v}$) for each feature was selected based on a heuristic analysis with windows between 200 ms and 2 s. Most dynamic features are commonly associated with the prior to the fall phase, or with the critical phase of the fall, which are estimated between 300–500 ms [27]. However, in this work, only $C_{10}$, $C_{11}$, and $C_{14}$ performed better with a window of $N_{v}=500$ ms. The other dynamic features improved with $N_{v}=1$ s (200 samples).

From Figure 3, features $C_{2}$ and $C_{8}$ obtained the higher accuracy once the filter was applied (95.0%±1.2% and 96.1%±0.75%, respectively). This result is consistent with the literature ([9], Table 1). In this case, $C_{2}$ would be preferred as it is static, i.e., it requires less memory and computational effort to be computed. The main difference between $C_{2}$ and the well known sum vector magnitude ($C_{1}$) is that it only includes the horizontal plane (*x*-axis and *z*-axis in our device). The position of the sensor in the center of mass of the body allows neglecting the vertical axis from the computation. With this, we reduce the number of false positives caused by the high accelerations achieved in the *y*-axis with many ADLs (walk, run, jump, etc.).

Regarding the other features, it is evident that not all of them improved their performance after filtering. Specifically, those based on integration behaved better without preprocessing, which is expected as they may reduce high frequency noise as a low-pass filter. Feature $C_{13}$, for example, achieved similar accuracy to $C_{2}$ without the need of implementing a digital filter. Selecting the best fitted feature would depend on the embedded device used and the way they are implemented. Finally, orientation and time based features presented an overall poor performance (comparable to the sum vector magnitude).

The inclusion of the filtering stage also defines the minimum allowed frequency sample. A preliminary analysis indicated that more elaborated filters or higher cut frequency values did not improve the accuracy. This result is meaningful as it suggests that a frequency sample of up to 11 Hz could be enough for fall detection (lower than any work in the literature), with its respective burden reduction. This gives an advantage to those features that performed better with the filter, given that the frequency sample is critical in wearable devices. This is because (*i*) the system remains more time in idle state; and (*ii*) more separation among samples allows more computations of the classifier. SisFall dataset was released with its original 200 Hz frequency sample, as a way to encourage other authors to obtain their own conclusions.

For illustrative purposes, in the remainder of this paper, we only show results of the five features that best performed: $C_{2}$, $C_{3}$, $C_{8}$, $C_{9}$, and $C_{13}$.

### 4.2. Training with Young vs. Elderly People

Our second proof-of-principle experiment accounted if training fall detection algorithms with young adults is adequate to use with elderly people. Table 5 shows sensitivity (SE), specificity (SP) and accuracy (AC) results after a 10-fold cross-validation performed only with data from young adults, and the results of using the obtained $T_{1}$ thresholds (included in Table 6) to test with data from elderly people.

The analysis presented mixed results. $C_{2}$, $C_{8}$ and $C_{13}$ lost performance while $C_{3}$ and $C_{9}$ even improved their accuracy (AC) when validated with elderly people. However, all features significantly reduced their sensitivity (SE, true positive rate). These results coincide with those presented in [8]. It is noteworthy, as there are clear differences among the participants of both studies. The SisFall dataset we release in this work is intended to help develop fall detection algorithms for healthy independent elderly people, while authors in [8] obtained their dataset with highly impaired institutionalized Parkinson’s patients.

The generalized variation in sensitivity and specificity (which increased in validation with elderly people) suggests a shift in all activities with respect to the threshold. We performed a second comparative analysis to determine if the threshold is better adjusted when the algorithms are trained exclusively with elderly people. Table 6 shows the validation accuracy of elderly people and threshold $T_{1}$ values from the previous analysis (test 1), and a new analysis training the algorithms only with elderly people (test 2). As a result, all features improved their performance with the new training (first two columns). Additionally, all features diminished their $T_{1}$ values, which confirms the shift between young and elderly people. This result makes evident the need of including data from elderly people in the training stage, especially because after training with elderly people, the accuracy was still below the one obtained with young people.

**Figure 4:**
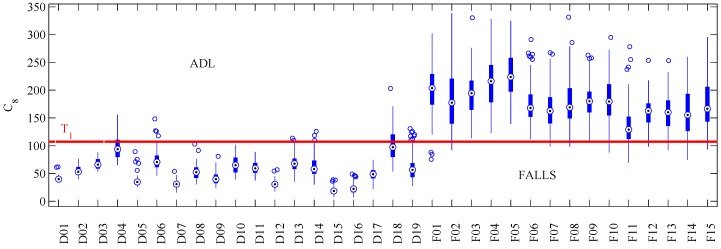
Maximum value per activity obtained with $C_{8}$. Most $T_{1}$ threshold crossings (horizontal **red** line) are contained in activities D04, D18 and F11.

A close review of individual activities of SisFall provided the following findings: (*i*) ADLs and falls simulated by elderly people were smaller in amplitude than those simulated by young people. Then, algorithms trained with data from young people tended to bias the thresholds upwards in amplitude; (*ii*) most features tended to fail in the same activities. Figure 4 shows box-plots of the maximum value obtained per activity with $C_{8}$ feature (with young adults group exclusively). Note that only few activities severely crossed the threshold (horizontal red line): jogging quickly (D04), jump (D18), and falling backward when trying to sit (F11).

During this study, we observed differences in the way young adults behaved and fell with respect to elderly people. As previously stated by [28], the dynamics of simulated (mimicked) and real-world falls can be different. They found that young people simulating falls tend to do it faster without trying to avoid the impact. This behavior can be observed in the videos released as Supplementary Materials of this paper [22]. On the other hand, because of his age and experience in Judo, the elderly person that performed falls always tried to cushion the hits, which is what we expect from someone having an undesired fall.

We acknowledge that our dataset only includes simulated falls of one elderly person (subject SE06), who also is a Judo expert (not representative of the population). However, it allowed us to obtain five controlled repetitions of 15 different types of falls (for a total of 75 falls). Authors in [8] obtained 29 real falls, but they did not release them and their population was also distant from independent elderly people. Additionally, they did not provide detailed information about each fall condition. SisFall is the first public dataset that includes ADLs from elderly people and falls from an elderly person.

### 4.3. Zero False Negatives

One way to increase the effectiveness of the fall detection algorithms consists of including a false alarm button, which allows the user to cancel ADL detected as falls (false positives) [29]. This method allows moving the threshold just below the minimum fall values (as $T_{2}$ does) [12]. Table 7 shows the specificity and accuracy obtained after a 10-fold cross-validation with all 38 subjects (sensitivity achieved approx. 99.99% ± 0.2% in all features). The loss of performance in all features is evident, failing in up to seven of every 10 ADL, and achieving only 84% of accuracy with $C_{9}$ (the best feature).

A fall detection system should not miss a single fall due to the medical implications every fall may carry on. Based on this statement, results with threshold $T_{2}$ may be more meaningful than with $T_{1}$. However, a failure rate of nearly 50/50 in ADL is prohibitive in real-life applications (the subject would be regularly pressing the false alarm button). The need of improving fall detection features stands, as a poor feature extraction requires more computationally intensive classifiers with the consequent battery life reduction [11].

## 5. Discussion

Research on elderly fall detection lacks public datasets with activities and falls simulated by elderly people. Available datasets have few activities and none include falls from the objective population. In this paper, we presented and made publicly available the SisFall dataset. It consisted of up to 34 activities (falls and ADLs) that were performed by 38 participants with a wearable device fixed to their waist. One of the participants was an elderly person that simulated both ADL and falls. Together with the dataset, we included videos of all simulated activities as an effort to help other researchers to replicate this work.

The SisFall dataset contains more participants, types of activities and recordings than any other publicly available dataset. It consists of 2706 ADL and 1798 falls, including data from 15 healthy independent elderly persons. To our knowledge, no public dataset contains data from elderly people, and their number of recordings is smaller (Mobifall: 342 ADLs and 288 falls; TFall: continuous ADLs and 240 falls; DLR: 961 ADLs and 56 falls; and Project Gravity: 138 ADLs and 72 falls).

We developed and released this dataset as a benchmark for other authors in the field. In that sense, we tested it with some of the most widely used features to detect falls, with three proof-of-principle experiments: the effect of the preprocessing stage, the importance of including data from elderly people, and how a threshold focused on maximum sensitivity severely reduces the specificity. Explanations about preprocessing are commonly simplified in most approaches available in the literature. Here, with a simple 4th order Butterworth filter, we increased the accuracy of several features. However, not all features improved their performance, which is expected as they share an integral-based nature; but it is a fact not previously discussed in the literature. Nevertheless, preprocessing is crucial in fall detection as it defines the minimum acquisition frequency, which, in this work, we found to be at 11 Hz for those features that indeed improved with the filtering stage.

In the second test, we analyzed the effect of training with young adults on a system developed to work with elderly people, which is usual in the field despite preliminary evidence that the results are biased [8]. Similar to this previous work, we found that the sensitivity is highly affected in all features once they are validated with the objective population. Note that Bagalà et al. [8] used 29 real falls of highly impaired Parkinson’s patients. In our case, we used 75 falls under controlled conditions from a single independent elderly person, which is also a martial arts expert. It is noteworthy that, despite the large difference among validation sets, our results presented the same trend of [8]. Moreover, when the classifiers were trained with elderly people, the accuracy was still lower than with young people. These findings suggest that, due to the overall higher acceleration that young people show in all activities, including ADLs, and falls from elderly people, it is crucial to obtain proper results. Additionally, the lower accuracy obtained when training with elderly people suggests that there is a need of a better feature extraction.

Developing a better feature extraction should be focused on specific activities. There are not many works focused on the types of falls elderly people suffer (most authors were limited to perform the same activities of previous works). However, the answers of our survey, previous works [5] and our findings suggest that if properly selected, authors could use a small sample of activities for their own tests. Performing an individual activity analysis (as presented in Figure 4) should help with the design of new features.

Our final test consisted of placing the threshold ($T_{2}$) below the fall value with minimum amplitude. In practice, fall detection systems are expected to detect all falls, while keeping the false positive rate as low as possible. Results of Table 7 presented poor results in all features. Note how a not too large increment in sensitivity caused significant reductions in specificity. This fact is noteworthy, as most works focus on maximizing accuracy instead of favoring fall detection. Authors that addressed this issue usually included a false-alarm button as part of their methodology [29].

Our dataset may be biased by two facts: (*i*) all of our falls were simulated (mimicked). Klenk et al. [28] stated that young people tend to fall faster than in real-life conditions; (*ii*) we only included falls from one independent elderly person; and, as a martial arts expert, this subject is not representative of the population. With respect to the first fact, the results of Section 4.2 show that training with young people effectively shows higher accelerations. However, this difference can be quantified and corrected by comparing their mean acceleration per activity versus the elderly subjects on the same dataset. About the second fact, our falls from an elderly person (Section 4.2) presented the same trend of a previous work that included real falls of impaired elderly people [8]. However, the crucial point here are the problems with obtaining real falls from healthy independent elderly people. In our case, we only had permission from the Ethics committee for simulating falls with one participant (SE06). Indeed, this participant always tried to soften the fall (as any person trying to avoid a fall would do). We consider that going farther with a wider and more realistic elderly fall dataset would be extremely challenging. Independent elderly people (the target of this work) fall on average once per year, i.e., to acquire a single fall would require a full year of continuous recording. Moreover, in this way, the actual conditions of the fall (activity, side of falling, etc.) may never be known. Despite these possible biases, we expect that this dataset will be a useful benchmark for other authors to test their own approaches and to solve the open issues presented in this work.

## 6. Conclusions

In this paper we presented and released SisFall, a fall and movement dataset acquired with 38 participants (15 of them elderly people). The data were acquired with an accelerometer fixed to their body. Along with this dataset we demonstrated that a 5 Hz fourth order filter keeps enough information for detecting falls on independent elderly people. Additionally, we showed that (as Bagalá et al. stated for institutionalized impaired elderly people) training fall detection algorithms with young people is not adequate for detecting falls on independent elderly people. The main problem found is that young people simulate falls and ADL with more acceleration than the expected with elderly people. Finally, we showed why finding maximum accuracy in fall detection algorithms is not a good measure for real-life applications, where the sensibility of the system must be fitted to detect falls, while reducing the false positive rate as possible. However, all tested features presented poor results with this requisite.

## Figures and Tables

**Table 1:** Types of falls selected for this work.

Code	Activity	Trials	Duration
F01	Fall forward while walking caused by a slip	5	15 s
F02	Fall backward while walking caused by a slip	5	15 s
F03	Lateral fall while walking caused by a slip	5	15 s
F04	Fall forward while walking caused by a trip	5	15 s
F05	Fall forward while jogging caused by a trip	5	15 s
F06	Vertical fall while walking caused by fainting	5	15 s
F07	Fall while walking, with use of hands in a table to dampen fall, caused by fainting	5	15 s
F08	Fall forward when trying to get up	5	15 s
F09	Lateral fall when trying to get up	5	15 s
F10	Fall forward when trying to sit down	5	15 s
F11	Fall backward when trying to sit down	5	15 s
F12	Lateral fall when trying to sit down	5	15 s
F13	Fall forward while sitting, caused by fainting or falling asleep	5	15 s
F14	Fall backward while sitting, caused by fainting or falling asleep	5	15 s
F15	Lateral fall while sitting, caused by fainting or falling asleep	5	15 s

**Table 2:** Types of activities of daily living selected for this work.

Code	Activity	Trials	Duration
D01	Walking slowly	1	100 s
D02	Walking quickly	1	100 s
D03	Jogging slowly	1	100 s
D04	Jogging quickly	1	100 s
D05	Walking upstairs and downstairs slowly	5	25 s
D06	Walking upstairs and downstairs quickly	5	25 s
D07	Slowly sit in a half height chair, wait a moment, and up slowly	5	12 s
D08	Quickly sit in a half height chair, wait a moment, and up quickly	5	12 s
D09	Slowly sit in a low height chair, wait a moment, and up slowly	5	12 s
D10	Quickly sit in a low height chair, wait a moment, and up quickly	5	12 s
D11	Sitting a moment, trying to get up, and collapse into a chair	5	12 s
D12	Sitting a moment, lying slowly, wait a moment, and sit again	5	12 s
D13	Sitting a moment, lying quickly, wait a moment, and sit again	5	12 s
D14	Being on one’s back change to lateral position, wait a moment, and change to one’s back	5	12 s
D15	Standing, slowly bending at knees, and getting up	5	12 s
D16	Standing, slowly bending without bending knees, and getting up	5	12 s
D17	Standing, get into a car, remain seated and get out of the car	5	25 s
D18	Stumble while walking	5	12 s
D19	Gently jump without falling (trying to reach a high object)	5	12 s

**Table 3:** Age, height and weight of the participants.

	Sex	Age	Height (m)	Weight (kg)
Elderly	Female	62–75	1.50–1.69	50–72
	Male	60–71	1.63–1.71	56–102
Adult	Female	19–30	1.49–1.69	42–63
	Male	19–30	1.65–1.83	58–81

**Table 4:** Feature extraction characteristics used to test the proposed dataset.

Type	Code	Feature	Equation
Amplitude	$C_{1}$	Sum vector magnitude	$C_{1}\left[k\right]=RMS\left(\tilde{a}\left[k\right]\right)=\sqrt{a_{x}^{2}\left[k\right]+a_{y}^{2}\left[k\right]+a_{z}^{2}\left[k\right]}$
	$C_{2}$	Sum vector magnitude on horizontal plane	$C_{2}\left[k\right]=\sqrt{a_{x}^{2}\left[k\right]+a_{z}^{2}\left[k\right]}$
	$C_{3}$	Maximum peak-to-peak acceleration amplitude	$C_{3}\left[k\right]=RMS\left(\max\left(\tilde{a}\left[k\right]\right)-\min\left(\tilde{a}\left[k\right]\right)\right)$
Orientation	$C_{4}$	Angle between *z*-axis and vertical	$C_{4}\left[k\right]=\mathrm{atan}2\left(\sqrt{{\left({\tilde{a}}_{x}\left[k\right]\right)}^{2}+{\left({\tilde{a}}_{z}\left[k\right]\right)}^{2}},-{\tilde{a}}_{y}\left[k\right]\right)$
	$C_{5}$	Orientation of person’s trunk	$C_{5}\left[k\right]=\sigma \left(\mathrm{atan}\left(\frac{RMS({\tilde{a}}_{x}\left[k\right],{\tilde{a}}_{z}\left[k\right])}{{\tilde{a}}_{y}\left[k\right]}\right)\right)$
	$C_{6}$	Orientation change in horizontal plane	$C_{6}\left[k\right]=\mathrm{mean}\left({\vec{a}}_{x}\left[k-N\right]\right)\cdot \mathrm{mean}\left({\vec{a}}_{x}\left[k\right]\right)$
Time	$C_{7}$	Jerk (rate of acceleration change)	$C_{7}\left[k\right]=\frac{{\vec{a}}_{x}\left[k\right]-{\vec{a}}_{x}\left[k-N\right]}{t[k]-t[k-N]}$
Statistics	$C_{8}$	Standard deviation magnitude on horizontal plane	$C_{8}\left[k\right]=\sqrt{\sigma_{x}^{2}\left[k\right]+\sigma_{z}^{2}\left[k\right]}$; with $\sigma_{i}=\mathrm{std}\left({\tilde{a}}_{i}\left[k\right]\right)$
	$C_{9}$	Standard deviation magnitude	$C_{9}\left[k\right]=\sqrt{\sigma_{x}^{2}\left[k\right]+\sigma_{y}^{2}\left[k\right]+\sigma_{z}^{2}\left[k\right]}$
Area	$C_{10}$	Signal magnitude area	$C_{10}\left[k\right]=\frac{1}{N}\left(\int |{\tilde{a}}_{x}\left[k\right]|dt+\int |{\tilde{a}}_{y}\left[k\right]|dt+\int |{\tilde{a}}_{z}\left[k\right]|dt\right)$
	$C_{11}$	Signal magnitude area on horizontal plane	$C_{11}\left[k\right]=\frac{1}{N}\left(\int |{\tilde{a}}_{x}\left[k\right]|dt+\int |{\tilde{a}}_{z}\left[k\right]|dt\right)$
	$C_{12}$	Activity signal magnitude area	$C_{12}\left[k\right]=\int \left(\sqrt{{\tilde{a}}_{x}^{2}\left[n\right]+{\tilde{a}}_{y}^{2}\left[n\right]+{\tilde{a}}_{z}^{2}\left[n\right]}\right)dn$
	$C_{13}$	Activity signal magnitude area on horizontal plane	$C_{13}\left[k\right]=\int \left(\sqrt{{\tilde{a}}_{x}^{2}\left[n\right]+{\tilde{a}}_{z}^{2}\left[n\right]}\right)dn$
	$C_{14}$	Velocity (approx.)	$C_{14}\left[k\right]=\frac{1}{N}\sqrt{{\left(\int {\tilde{a}}_{x}\left[k\right]dt\right)}^{2}+{\left(\int {\tilde{a}}_{z}\left[k\right]dt\right)}^{2}}$

**Table 5:** Sensitivity (SE), specificity (SP) and accuracy (AC) after training with young adults and validating either with young adults or elderly people.

Feature	Young			Elderly		
	SE	SP	AC	SE	SP	AC
$C_{2}$	**94.28**	96.13	95.21	**77.33**	97.67	87.49
$C_{3}$	**98.53**	80.50	89.51	**84.00**	96.42	90.21
$C_{8}$	**95.54**	96.38	95.96	**85.33**	98.10	91.72
$C_{9}$	**97.79**	80.70	89.25	**88.00**	96.42	92.21
$C_{13}$	**92.56**	94.41	93.49	**62.67**	95.19	78.93

**Table 6:** Variation in accuracy and threshold $T_{1}$ after training exclusively with the young but validating with elderly people (test 1), and then training and validating with elderly people (test 2).

Feature	AC (%) with Elderly		Threshold $T_{1}$	
	Test 1	Test 2	Test 1	Test 2
$C_{2}$	87.49	90.45 ± 5.89	1.07 ± 0.029	0.97 ± 0.012
$C_{3}$	90.21	90.85 ± 7.25	1.48 ± 0.017	1.23 ± 0.024
$C_{8}$	91.72	92.36 ± 6.80	0.40 ± 0.004	0.36 ± 0.003
$C_{9}$	92.21	92.58 ± 7.10	0.43 ± 0.009	0.36 ± 0.002
$C_{13}$	78.93	80.73 ± 5.62	0.08 ± 9.35 × $10^{-5}$	0.07 ± 0.002

**Table 7:** Specificity (SP) and accuracy (AC) after testing data from all subjects with threshold $T_{2}$.

Feature	SP	AC
$C_{2}$	32.97 ± 6.46	66.43 ± 3.06
$C_{3}$	59.04 ± 5.56	79.49 ± 2.70
$C_{8}$	38.34 ± 5.58	69.14 ± 2.71
$C_{9}$	67.97 ± 2.86	**83.96 ± 1.37**
$C_{13}$	37.80 ± 3.42	68.88 ± 1.69

## Supplementary Materials

The following files are available online [22]:

- **SisFall movement and fall dataset.** Text files with all recorded activities and a Readme with particular information of all subjects and recordings.
- **Video recordings of all activities.** Each activity included in the SisFall dataset was video recorded and included in this material.
- **Tables and figures with results of all features.** The same experiments shown along the paper with only five features were performed with the 14 selected for this work.
